# Supplementary material for: HIVprotI: an integrated web based platform for prediction and design of HIV proteins inhibitors
Source: J Cheminform. 2018 Mar 9;10:12. doi: 10.1186/s13321-018-0266-y (PMC5845081; doi:10.1186/s13321-018-0266-y)
Supplement: Supplementary file 2 — Additional file 2. Source code of HIVProtI web server. [file 13321_2018_266_MOESM2_ESM.zip › HIVprotI_Source-code/hivprotI/weka-3-6-6/doc/weka/gui/sql/SqlViewer.html]

SqlViewer


---


|  |  |  |
| --- | --- | --- |
| **Overview** \| **Package** \| **Class** \| **Tree** \| **Deprecated** \| **Index** \| **Help** \| **Weka's home** | |  |
| **PREV CLASS**   **NEXT CLASS** | **FRAMES**    **NO FRAMES**     **All Classes** |  |
| SUMMARY: NESTED \| FIELD \| CONSTR \| METHOD | DETAIL: FIELD \| CONSTR \| METHOD |  |


---


## weka.gui.sql Class SqlViewer

```
java.lang.Object
  java.awt.Component
      java.awt.Container
          javax.swing.JComponent
              javax.swing.JPanel
                  weka.gui.sql.SqlViewer
```

**All Implemented Interfaces:**: java.awt.image.ImageObserver, java.awt.MenuContainer, java.io.Serializable, java.util.EventListener, javax.accessibility.Accessible, ConnectionListener, HistoryChangedListener, QueryExecuteListener, ResultChangedListener

---

``` public class SqlViewer extends javax.swing.JPanel implements ConnectionListener, HistoryChangedListener, QueryExecuteListener, ResultChangedListener ```

Represents a little tool for querying SQL databases.

**Version:**
:   $Revision: 7059 $

**Author:**
:   FracPete (fracpete at waikato dot ac dot nz)

**See Also:**: Serialized Form

---

**Nested Class Summary**

**Nested classes/interfaces inherited from class javax.swing.JComponent**

`javax.swing.JComponent.AccessibleJComponent`

**Nested classes/interfaces inherited from class java.awt.Component**

`java.awt.Component.BaselineResizeBehavior`


| **Field Summary** | |
| --- | --- |
| `static java.lang.String` | `HEIGHT`             the height property in the history file. |
| `static java.lang.String` | `WIDTH`             the width property in the history file. |

**Fields inherited from class javax.swing.JComponent**

`TOOL_TIP_TEXT_KEY, UNDEFINED_CONDITION, WHEN_ANCESTOR_OF_FOCUSED_COMPONENT, WHEN_FOCUSED, WHEN_IN_FOCUSED_WINDOW`

**Fields inherited from class java.awt.Component**

`BOTTOM_ALIGNMENT, CENTER_ALIGNMENT, LEFT_ALIGNMENT, RIGHT_ALIGNMENT, TOP_ALIGNMENT`

**Fields inherited from interface java.awt.image.ImageObserver**

`ABORT, ALLBITS, ERROR, FRAMEBITS, PROPERTIES, SOMEBITS`


**Constructor Summary**

`SqlViewer(javax.swing.JFrame parent)`
  
          initializes the SqlViewer.


| **Method Summary** | |
| --- | --- |
| `void` | `addConnectionListener(ConnectionListener l)`             adds the given listener to the list of listeners. |
| `void` | `addHistoryChangedListener(HistoryChangedListener l)`             adds the given listener to the list of listeners. |
| `void` | `addQueryExecuteListener(QueryExecuteListener l)`             adds the given listener to the list of listeners. |
| `void` | `addResultChangedListener(ResultChangedListener l)`             adds the given listener to the list of listeners. |
| `void` | `clear()`             calls the clear method of all sub-panels to set back to default values and free up memory. |
| `void` | `connectionChange(ConnectionEvent evt)`             This method gets called when the connection is either established or disconnected. |
| `java.lang.String` | `getPassword()`             returns the password from the currently active tab in the ResultPanel, otherwise an empty string. |
| `java.lang.String` | `getQuery()`             returns the query from the currently active tab in the ResultPanel, otherwise an empty string. |
| `java.lang.String` | `getURL()`             returns the database URL from the currently active tab in the ResultPanel, otherwise an empty string. |
| `java.lang.String` | `getUser()`             returns the user from the currently active tab in the ResultPanel, otherwise an empty string. |
| `void` | `historyChanged(HistoryChangedEvent evt)`             This method gets called when a history is modified. |
| `static void` | `main(java.lang.String[] args)`             starts the SQL-Viewer interface. |
| `void` | `queryExecuted(QueryExecuteEvent evt)`             This method gets called when a query has been executed. |
| `void` | `removeConnectionListener(ConnectionListener l)`             removes the given listener from the list of listeners. |
| `void` | `removeHistoryChangedListener(HistoryChangedListener l)`             removes the given listener from the list of listeners. |
| `void` | `removeQueryExecuteListener(QueryExecuteListener l)`             removes the given listener from the list of listeners. |
| `void` | `removeResultChangedListener(ResultChangedListener l)`             removes the given listener from the list of listeners. |
| `void` | `resultChanged(ResultChangedEvent evt)`             This method gets called when a query has been executed. |
| `void` | `saveSize()`             obtains the size of the panel and saves it in the history. |

**Methods inherited from class javax.swing.JPanel**

`getAccessibleContext, getUI, getUIClassID, setUI, updateUI`

**Methods inherited from class javax.swing.JComponent**

`addAncestorListener, addNotify, addVetoableChangeListener, computeVisibleRect, contains, createToolTip, disable, enable, firePropertyChange, firePropertyChange, firePropertyChange, getActionForKeyStroke, getActionMap, getAlignmentX, getAlignmentY, getAncestorListeners, getAutoscrolls, getBaseline, getBaselineResizeBehavior, getBorder, getBounds, getClientProperty, getComponentPopupMenu, getConditionForKeyStroke, getDebugGraphicsOptions, getDefaultLocale, getFontMetrics, getGraphics, getHeight, getInheritsPopupMenu, getInputMap, getInputMap, getInputVerifier, getInsets, getInsets, getListeners, getLocation, getMaximumSize, getMinimumSize, getNextFocusableComponent, getPopupLocation, getPreferredSize, getRegisteredKeyStrokes, getRootPane, getSize, getToolTipLocation, getToolTipText, getToolTipText, getTopLevelAncestor, getTransferHandler, getVerifyInputWhenFocusTarget, getVetoableChangeListeners, getVisibleRect, getWidth, getX, getY, grabFocus, isDoubleBuffered, isLightweightComponent, isManagingFocus, isOpaque, isOptimizedDrawingEnabled, isPaintingForPrint, isPaintingTile, isRequestFocusEnabled, isValidateRoot, paint, paintImmediately, paintImmediately, print, printAll, putClientProperty, registerKeyboardAction, registerKeyboardAction, removeAncestorListener, removeNotify, removeVetoableChangeListener, repaint, repaint, requestDefaultFocus, requestFocus, requestFocus, requestFocusInWindow, resetKeyboardActions, reshape, revalidate, scrollRectToVisible, setActionMap, setAlignmentX, setAlignmentY, setAutoscrolls, setBackground, setBorder, setComponentPopupMenu, setDebugGraphicsOptions, setDefaultLocale, setDoubleBuffered, setEnabled, setFocusTraversalKeys, setFont, setForeground, setInheritsPopupMenu, setInputMap, setInputVerifier, setMaximumSize, setMinimumSize, setNextFocusableComponent, setOpaque, setPreferredSize, setRequestFocusEnabled, setToolTipText, setTransferHandler, setVerifyInputWhenFocusTarget, setVisible, unregisterKeyboardAction, update`

**Methods inherited from class java.awt.Container**

`add, add, add, add, add, addContainerListener, addPropertyChangeListener, addPropertyChangeListener, applyComponentOrientation, areFocusTraversalKeysSet, countComponents, deliverEvent, doLayout, findComponentAt, findComponentAt, getComponent, getComponentAt, getComponentAt, getComponentCount, getComponents, getComponentZOrder, getContainerListeners, getFocusTraversalKeys, getFocusTraversalPolicy, getLayout, getMousePosition, insets, invalidate, isAncestorOf, isFocusCycleRoot, isFocusCycleRoot, isFocusTraversalPolicyProvider, isFocusTraversalPolicySet, layout, list, list, locate, minimumSize, paintComponents, preferredSize, printComponents, remove, remove, removeAll, removeContainerListener, setComponentZOrder, setFocusCycleRoot, setFocusTraversalPolicy, setFocusTraversalPolicyProvider, setLayout, transferFocusBackward, transferFocusDownCycle, validate`

**Methods inherited from class java.awt.Component**

`action, add, addComponentListener, addFocusListener, addHierarchyBoundsListener, addHierarchyListener, addInputMethodListener, addKeyListener, addMouseListener, addMouseMotionListener, addMouseWheelListener, bounds, checkImage, checkImage, contains, createImage, createImage, createVolatileImage, createVolatileImage, dispatchEvent, enable, enableInputMethods, firePropertyChange, firePropertyChange, firePropertyChange, firePropertyChange, firePropertyChange, getBackground, getBounds, getColorModel, getComponentListeners, getComponentOrientation, getCursor, getDropTarget, getFocusCycleRootAncestor, getFocusListeners, getFocusTraversalKeysEnabled, getFont, getForeground, getGraphicsConfiguration, getHierarchyBoundsListeners, getHierarchyListeners, getIgnoreRepaint, getInputContext, getInputMethodListeners, getInputMethodRequests, getKeyListeners, getLocale, getLocation, getLocationOnScreen, getMouseListeners, getMouseMotionListeners, getMousePosition, getMouseWheelListeners, getName, getParent, getPeer, getPropertyChangeListeners, getPropertyChangeListeners, getSize, getToolkit, getTreeLock, gotFocus, handleEvent, hasFocus, hide, imageUpdate, inside, isBackgroundSet, isCursorSet, isDisplayable, isEnabled, isFocusable, isFocusOwner, isFocusTraversable, isFontSet, isForegroundSet, isLightweight, isMaximumSizeSet, isMinimumSizeSet, isPreferredSizeSet, isShowing, isValid, isVisible, keyDown, keyUp, list, list, list, location, lostFocus, mouseDown, mouseDrag, mouseEnter, mouseExit, mouseMove, mouseUp, move, nextFocus, paintAll, postEvent, prepareImage, prepareImage, remove, removeComponentListener, removeFocusListener, removeHierarchyBoundsListener, removeHierarchyListener, removeInputMethodListener, removeKeyListener, removeMouseListener, removeMouseMotionListener, removeMouseWheelListener, removePropertyChangeListener, removePropertyChangeListener, repaint, repaint, repaint, resize, resize, setBounds, setBounds, setComponentOrientation, setCursor, setDropTarget, setFocusable, setFocusTraversalKeysEnabled, setIgnoreRepaint, setLocale, setLocation, setLocation, setName, setSize, setSize, show, show, size, toString, transferFocus, transferFocusUpCycle`

**Methods inherited from class java.lang.Object**

`equals, getClass, hashCode, notify, notifyAll, wait, wait, wait`

**Field Detail**

### WIDTH

```
public static final java.lang.String WIDTH
```

:   the width property in the history file.

    **See Also:**: Constant Field Values

---


### HEIGHT

```
public static final java.lang.String HEIGHT
```

:   the height property in the history file.

    **See Also:**: Constant Field Values


**Constructor Detail**

### SqlViewer

```
public SqlViewer(javax.swing.JFrame parent)
```

:   initializes the SqlViewer.

    **Parameters:**: `parent` - the parent of this panel


**Method Detail**

### connectionChange

```
public void connectionChange(ConnectionEvent evt)
```

:   This method gets called when the connection is either established
    or disconnected.

    :   **Specified by:**: `connectionChange` in interface `ConnectionListener`
    :   **Parameters:**: `evt` - the event

---


### queryExecuted

```
public void queryExecuted(QueryExecuteEvent evt)
```

:   This method gets called when a query has been executed.

    :   **Specified by:**: `queryExecuted` in interface `QueryExecuteListener`
    :   **Parameters:**: `evt` - the event

---


### resultChanged

```
public void resultChanged(ResultChangedEvent evt)
```

:   This method gets called when a query has been executed.

    :   **Specified by:**: `resultChanged` in interface `ResultChangedListener`
    :   **Parameters:**: `evt` - the event

---


### historyChanged

```
public void historyChanged(HistoryChangedEvent evt)
```

:   This method gets called when a history is modified.
    It saves the history immediately to the users home directory.

    :   **Specified by:**: `historyChanged` in interface `HistoryChangedListener`
    :   **Parameters:**: `evt` - the event

---


### saveSize

```
public void saveSize()
```

:   obtains the size of the panel and saves it in the history.

    :   **See Also:**: `saveHistory()`

---


### clear

```
public void clear()
```

:   calls the clear method of all sub-panels to set back to default values
    and free up memory.

---


### getURL

```
public java.lang.String getURL()
```

:   returns the database URL from the currently active tab in the ResultPanel,
    otherwise an empty string.

    :   **Returns:**: the currently selected tab's URL **See Also:**: `ResultPanel`

---


### getUser

```
public java.lang.String getUser()
```

:   returns the user from the currently active tab in the ResultPanel,
    otherwise an empty string.

    :   **Returns:**: the currently selected tab's user **See Also:**: `ResultPanel`

---


### getPassword

```
public java.lang.String getPassword()
```

:   returns the password from the currently active tab in the ResultPanel,
    otherwise an empty string.

    :   **Returns:**: the currently selected tab's password **See Also:**: `ResultPanel`

---


### getQuery

```
public java.lang.String getQuery()
```

:   returns the query from the currently active tab in the ResultPanel,
    otherwise an empty string.

    :   **Returns:**: the currently selected tab's query **See Also:**: `ResultPanel`

---


### addConnectionListener

```
public void addConnectionListener(ConnectionListener l)
```

:   adds the given listener to the list of listeners.

    :   **Parameters:**: `l` - the listener to add to the list

---


### removeConnectionListener

```
public void removeConnectionListener(ConnectionListener l)
```

:   removes the given listener from the list of listeners.

    :   **Parameters:**: `l` - the listener to remove

---


### addQueryExecuteListener

```
public void addQueryExecuteListener(QueryExecuteListener l)
```

:   adds the given listener to the list of listeners.

    :   **Parameters:**: `l` - the listener to add to the list

---


### removeQueryExecuteListener

```
public void removeQueryExecuteListener(QueryExecuteListener l)
```

:   removes the given listener from the list of listeners.

    :   **Parameters:**: `l` - the listener to remove

---


### addResultChangedListener

```
public void addResultChangedListener(ResultChangedListener l)
```

:   adds the given listener to the list of listeners.

    :   **Parameters:**: `l` - the listener to add to the list

---


### removeResultChangedListener

```
public void removeResultChangedListener(ResultChangedListener l)
```

:   removes the given listener from the list of listeners.

    :   **Parameters:**: `l` - the listener to remove

---


### addHistoryChangedListener

```
public void addHistoryChangedListener(HistoryChangedListener l)
```

:   adds the given listener to the list of listeners.

    :   **Parameters:**: `l` - the listener to add to the list

---


### removeHistoryChangedListener

```
public void removeHistoryChangedListener(HistoryChangedListener l)
```

:   removes the given listener from the list of listeners.

    :   **Parameters:**: `l` - the listener to remove

---


### main

```
public static void main(java.lang.String[] args)
```

:   starts the SQL-Viewer interface.

    :   **Parameters:**: `args` - the commandline arguments - ignored


---


|  |  |  |
| --- | --- | --- |
| **Overview** \| **Package** \| **Class** \| **Tree** \| **Deprecated** \| **Index** \| **Help** \| **Weka's home** | |  |
| **PREV CLASS**   **NEXT CLASS** | **FRAMES**    **NO FRAMES**     **All Classes** |  |
| SUMMARY: NESTED \| FIELD \| CONSTR \| METHOD | DETAIL: FIELD \| CONSTR \| METHOD |  |


---
